# Doxycycline treatment in dialysis related amyloidosis: discrepancy between antalgic effect and inflammation, studied with FDG-positron emission tomography: a case report

**DOI:** 10.1186/s12882-017-0698-z

**Published:** 2017-09-06

**Authors:** Giorgina Barbara Piccoli, Mammar Hachemi, Ida Molfino, Jean Philippe Coindre, Charles Boursot

**Affiliations:** 1 Dipartimento di Scienze Cliniche e Biologiche, Università di Torino, Turin, Italy; 2 Nuclear Medicine Centre Hospitalier Le Mans, 72000 Le Mans, France; 3 Nephrologie, Centre Hospitalier du Mans, 198 Avenue Roubillard, 72000 Le Mans, France

**Keywords:** Dialysis related amyloidosis, Hemodialysis, Hemodiafiltration, Long-term survival, Positron emission tomography, Doxycycline

## Abstract

**Background:**

No effective treatment is currently available and dialysis related amyloidosis continues to be invalidating in long-term dialysis patients. A recent case series reported reduction of osteoarticular pain on doxycycline treatment, extending the indications of this drug, used in other uncommon forms of amyloidosis, to dialysis patients. Explanations of the antalgic effect were the anti-inflammatory properties and anti-coiling effects of tetracycline.

**Case presentation:**

Our report regards a 54-year-old woman, who was never transplanted and has been on hemodialysis and hemodiafiltration for overall 37 years, due to renal hypoplasia. In spite of high efficiency hemodiafiltration, she complained of increasing, invalidating osteoarticular pain; history and imaging suggested beta-2 microglobulin amyloid. Positron emission tomography (PET scan) identified metabolically active lesions in the involved settings. Low-dose doxycycline (100 mg/day) was started, leading to a considerable decrease in pain (over 6 months, from 7 to 8 to 4–5 on a 0–10 scale). At 6 months, a PET scan showed unmodified or increased uptake in the involved settings.

**Conclusions:**

In summary, the previously described antalgic effect of doxycycline in dialysis related amyloidosis is confirmed in our case, the first studied using PET scan. The pattern at PET can suggests that the antalgic effect is independent from inflammation and points to other factors, such as interaction with fibril geometry or with bone structure.

## Background

Dialysis related amyloidosis is a long-term complication of all forms of dialysis and continues to be invalidating in patients on long-term dialysis [1–5].

This form of systemic amyloidosis is linked to the deposition of amyloid fibril principally composed of beta-2 microglobuline, a non-variable chain of MHC class I membrane protein, ubiquitous on the surface of nucleated cells. Every day, 2–4 mg/kg of Beta 2 microglobuline are physiologically cleared by the kidney; the clearance decreased progressively in chronic kidney disease and the molecule is at best incompletely cleared by dialysis, since a positive balance is even the most permeable membranes [1–5].

Fibril deposition may occur in any organ, and its presence may be diffuse or mimic neoplastic diseases; however, the osteoarticular system is almost invariably involved, due to the high affinity of beta 2 microglobulin for collagen and dialysis related amyloidosis is the main cause of carpal tunnel syndrome and scapula–humeral periarthritis in dialysis patients. Even if some deposits of Beta 2 microglobulin may be found in virtually all patients treated by dialysis for at least 15 years, the correlation between entity of deposits and functional derangement is incomplete, the clinical manifestations vary widely, and this variation is only partially understood [4, 5].

Efficient dialysis can reduce the speed at which beta-2 microglobulin accumulates, and thus is probably the main reason for the decrease in the incidence of dialysis related amyloidosis with respect to the past, although it has not completely disappeared, in particular in patients who were never grafted [7–9]. The Japanese experience is particularly important, because of the low penetrance of kidney transplantation in this country, for clinical, cultural and logistic reasons. Although Japanese-led experiences, with specific absorption columns, able to effectively remove amyloid fibres, are promising, the devices are extremely expensive and not widely available [10–12].

Amyloid deposits elicit an important chronic inflammatory response, and this is an important element in the development of osteoarticular damage; as it will be further discussed, the choice of positron emission tomography (PET scan) for monitoring our patient is based upon its capacity to detect the inflammatory response elicited by the amyloid deposits more then the deposits themselves [1–5].

Many questions remain open, such as the role of precursors proteins, the relationship between circulating levels of Beta-2 microglobulin, the facilitating role of inflammation and the reversibility after successful kidney transplantation [4–7].

Due also to the fact that dialysis related amyloidosis is a protean disease, a precise diagnostic scoring, and, above all, an agreed treatment are not available so-far [13–15]. In spite of its different manifestations, osteoarticular pain is the main, almost universal, complaint [13–15].

An interesting recent case series reported significant reduction of osteoarticular pain on doxycycline treatment, potentially extending the indications of this drug, used with relevant clinical advantages in other uncommon forms of amyloidosis, to dialysis patients [16–21]. The case series reports a persistent beneficial effect on pain and on the ability to do their daily chores in three patients treated with low-dose doxycycline for up to 1 year [16]. The reasons for this effect are not clear: in fact, the amyloid tissue was stable at magnetic resonance analysis, and an anti-coiling effect and coating of the amyloid tissue have been postulated, since a direct antalgic effect seemed improbable, as no such benefit had been noted in other inflammatory diseases [16]., On the basis of this one favourable report on the safety and efficacy of low-dose doxycycline in dialysis related amyloidosis, after obtaining informed consent, we started treatment in a 54-year-old patient with congenital renal hypoplasia, who chose not to be transplanted and has been on dialysis for 37 years. This is the report of her clinical evolution and of her imaging data, the first study of a patient treated with doxycycline for dialysis-related amyloidosis, described with positron emission tomography (PET scan), a technique increasingly used in detecting and evaluating amyloidosis [22–26].

## Case presentation

Our patient is a 54-year-old Caucasian woman with congenital renal hypoplasia, who chose not to be transplanted and has been on dialysis for 37 years.

Her dialysis treatment started on acetate dialysis, followed by bicarbonate dialysis, and hemodiafultration was performed in the last 25 years, with high surface, high flux dialysers, in keeping with the policies of the French school [27, 28].

During her many years on dialysis, she underwent partial paratyroidectomy in 1989 and reintervention 10 years later; she suffered from carpal tunnel syndrome, one of the main markers of dialysis related amyloidosis, and surgery was performed in 1999 and in 2002. She also underwent a partial mastectomy for the presence of an in situ carcinoma in 2003, cholecystectomy in 2010 and excision of sigmoid polyps in 2013.

In spite of good dialysis efficiency and tolerance on thrice weekly, high-flux hemodiafiltration, the presence of increasingly severe osteoarticular pain, with progressive reduction of motility mainly of the shoulders and hips, has been a growing complaint in the last 10 years. Carpal tunnel surgery suggested the presence of clinically evident beta-2 microglobulin related amyloidosis for at least 15 years.

We chose PET scan for following our patient on account of its potential to evaluate the inflammatory component, which we believed was at least partially related to pain; our initial hypothesis was that a reduction of inflammation could explain the antalgic effect of doxycycline [16, 18, 25].

**Fig. 1:**
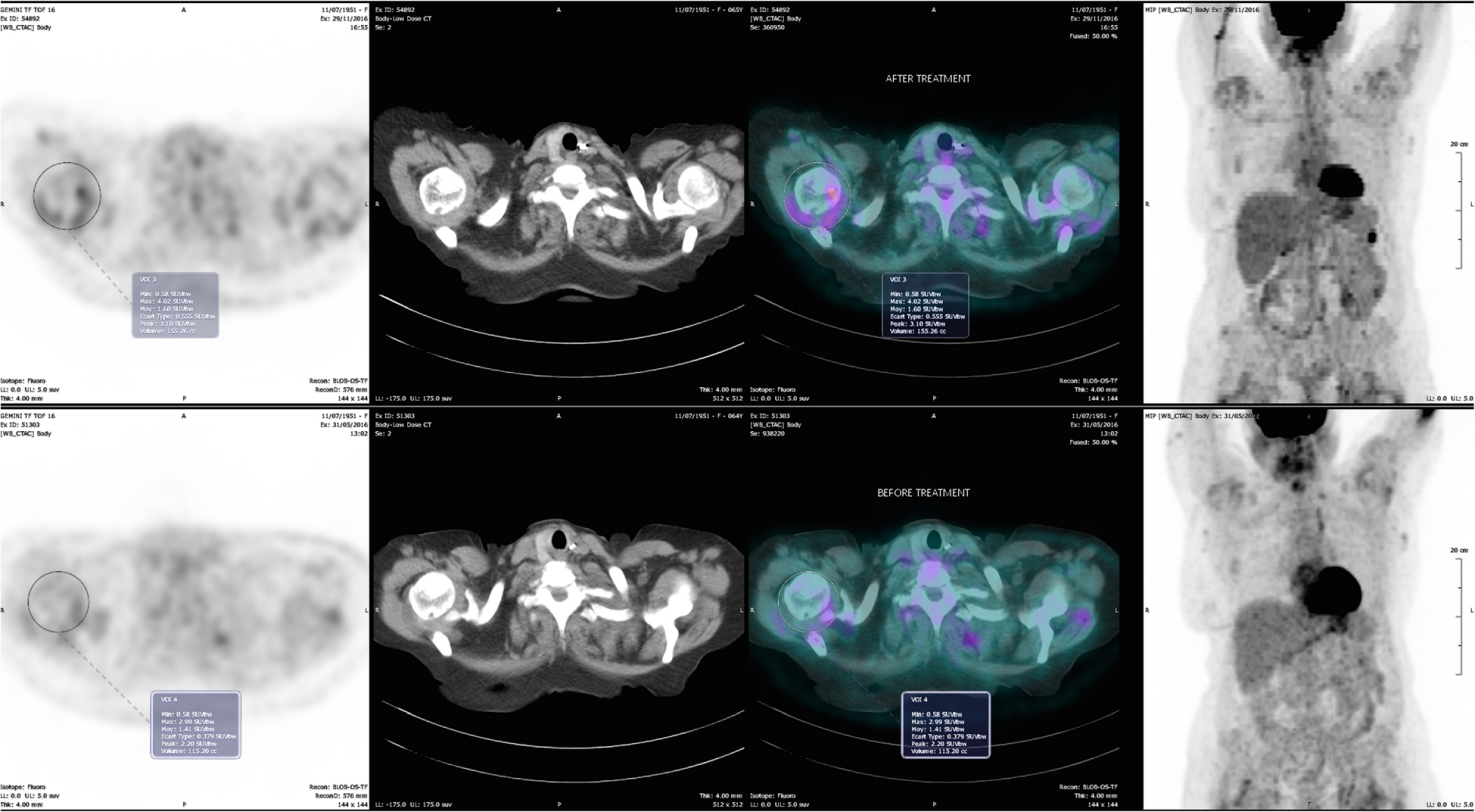
PET scan: upper image: after treatment; lower: before treatment. Hips. Diffuse, metabolically active deposits, specifically in the settings of intense pain. The pattern is consistent with diffuse periarticular hyperfixation. The metabolic activity is moderate, the standardised uptake value (SUV) increases from 3.40 to 4.19 at 6 months

**Fig. 2:**
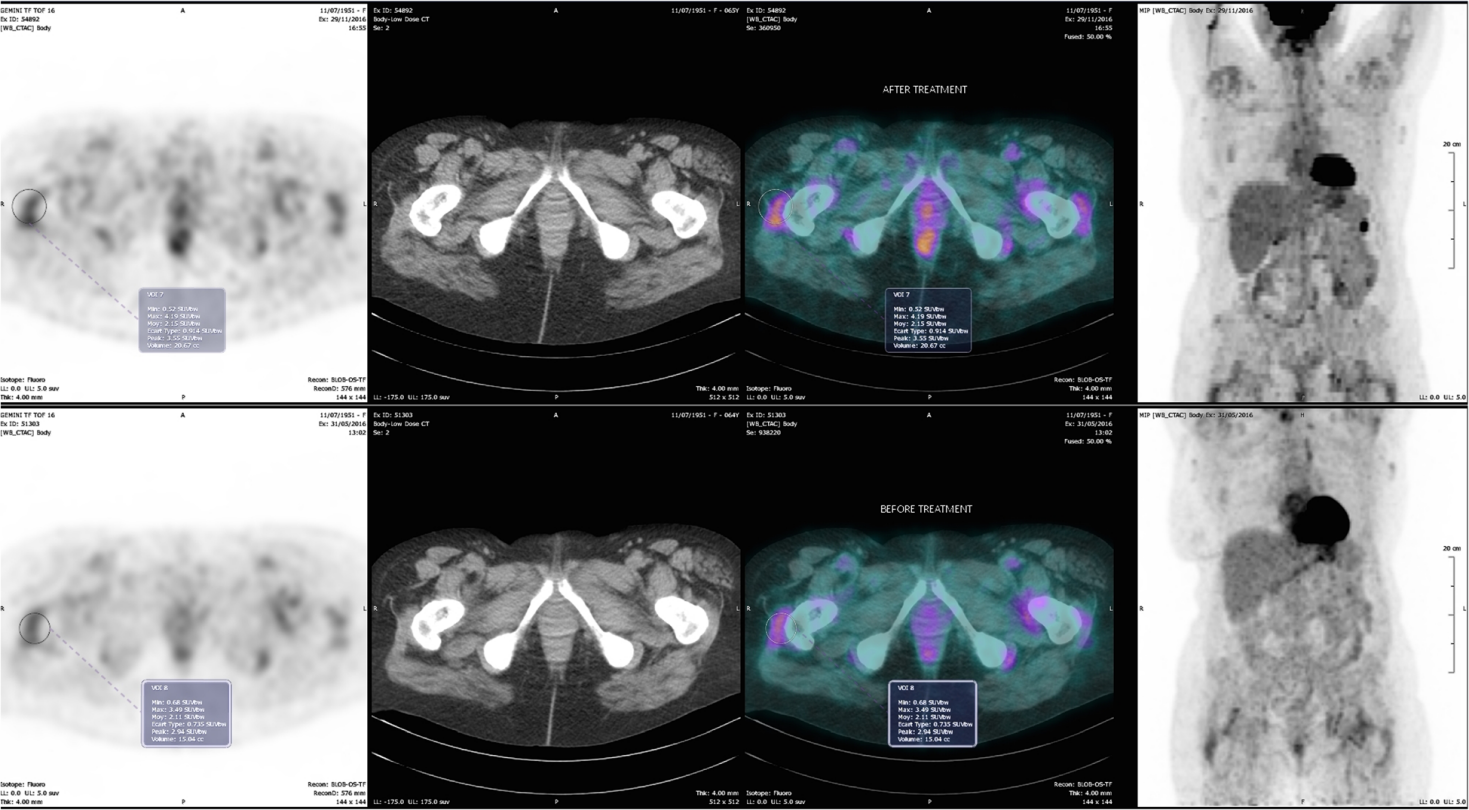
PET scan: upper image: after treatment; lower: before treatment. Shoulders. Diffuse, metabolically active deposits, specifically in the settings of pain. The pattern is consistent with diffuse periarticular hyperfixation. The SUV increases from 2.99 to 4.02 at 6 months

The image shown in Figs. 1 and 2, suggests the presence of diffuse, metabolically active deposits, specifically in the settings of intense pain (hips and shoulders). The pattern is consistent with diffuse periarticular hyperfixation on shoulders and hips. The metabolic activity is moderate, as witnessed by a standardised uptake value (SUV) of 2.99 on shoulders and 3.40 on hips.

Low-dose doxycycline (100 mg at lunchtime) was started, with good tolerance and no side effects; no other drugs/medications were changed during this period. The patient’s usual treatment consisted in 1 g/day of calcium carbonate; 0.5 mcg/day of 1–25 OH vitamin D; 20 mg/day of folic acid; 100,000 units/month of 25-OH vitamin D; 20 mg/day of esomeprazole; 25 mg/day of amitriptyline; up to 4 g/day of paracetamol in case of pain. The patient’s dialysis schedule was not modified (hemodiafiltration, 4 h three times per week, blood flow 300 mL/min, dialysate flow 700 ml/min; highly permeable membrane 2.1 m2) and dialysis efficiency was stable (range during the period: KtV Daugirdas-2: 1.65–1.80; normalised-PCR: 0.9–1.0 g/Kg/day). Anemia was corrected at the target (haemoglobin 11.3–12.2 g/dL, as was acidosis, with predialysis HCO3 22–24 mEq/L and the patient’s calcium/phosphate balance was acceptable, with moderate hyperparathyroidism (PTH ranged from 250 to 520 pg/mL)). No other signs of inflammation were present, with a normal C-reactive protein at 5/6 monthly biochemical profiles, while the relatively low albumin levels (3.0–3.3 g/dL, were interpreted as linked to the chronic losses on high flux hemodiafiltration).

Within 1 month from the start of treatment with doxycycline the patient reported initial pain relief (from 7 to 8 to 6–7 on a 0–10 analogic scale), which persisted and further improved at 6 months (decreasing to 4–5 on a the same scale). Consequently, she was able to reduce her doses of antalgic medication from about 4 to 1 g/day of paractetamol and to almost entirely discontinue other occasional pain relievers.

Interestingly, a second PET scan, performed 6 months after the start of doxycycline, was showed an increase in metabolic activity on the peri-articular level, in the absence of relevant metabolic changes, and without any increase in acute phase reactants. The SUV increased from 2.99 to 4.02 on shoulders and from 3.40 to 4.19 on hips (Figs. 1, 2).

## Discussion

To the best of our knowledge, this case is the fourth one employing doxycycline treatment in dialysis related amyloidosis to be reported in the literature, and is the first one studied using PET scan. While dealing with a combination of rare disease and unconventional treatments, on the account of the role of case reports as hypothesis generators, it may offer three interesting occasions for discussion.

The first is that it confirms the antalgic effect of doxycycline in dialysis related amyloidosis, in keeping with the previous report by Montagna et al., which suggested that this low-cost, well tolerated treatment, which had been successfully employed in other rare forms of amyloidosis, would be suitable in treating dialysis related amyloidosis [16].

Our patient, who had a long history of end-stage renal disease, treated exclusively by dialysis, had had clinical signs of amyloidosis (carpal tunnel) for at least 15 years, and relevant osteoarticular pain. To quantify pain reduction we employed the same analogic 0–10 pain scale used in the original report, and were able to confirm the same pattern reported in the previous three cases, i.e. a progressive decrease in pain starting in the first month of treatment, with further improvement at 6 months; interestingly the decrease is the same as what was reported in the original cases 1 and 3 [16]. Tolerance has been very good, and treatment is continuing.

The second point of interest is the relevance of PET scan in the functional definition of amyloid deposits, in keeping with an increasing number of reports on its use in other forms of amyloidosis and with preliminary data in dialysis related amyloidosis [22–25]. The potential advantage of PET scan is the lack of known toxicity of fluorodexoxyglucose (FDG) in dialysis patients, differently from other contrast media, in particular those containing gadolinium [25–29]. In our case, an added value was to allow detection of possible recurrences or metastases of her breast cancer, an advantage that could be relevant also in other patients, given the increased incidence of neoplastic diseases in patients on long-term renal replacement therapy [30–32].

The third point is related to the interpretation of the increase in the hypermetabolic response detected by PET scan after 6 months of treatment, despite progressive, persistent reduction in osteoarticular pain (Fig. 2). In fact, as stated in the report by Montagna et al., the reason for the effect of doxycycline is not clear, and does not seem to be related to an evident reduction in deposits, as has been observed in other forms of amyloidosis [16].

Our case may add information from the functional point of view, considering the sensitivity of FDG-PET for metabolically active tissues: our finding suggests that the effect is not linked to a reduction in inflammation, which is still present, or may even have increased in settings where amyloid is deposited (Fig. 2). Consequently, our case could open the way to other hypotheses: the antalgic benefit obtained may be due to subtle structural changes in amyloid geometry or in bone composition, on account of the interactions between doxycycline and amyloid fibrils and the fixation of doxycycline in the bones, potentially preventing bone loss [31–34]. Furthermore, the enhancement of the inflammatory response may be due to the recruitment of inflammatory cells, such as macrophages, that may be able to slowly decrease deposits by catabolising the amyloid fibrils [35, 36].

## Conclusion

Overall, our case emphasizes the importance of dialysis related amyloidosis, a disease that is sill present in the dialysis population and deserves attention, given its profound impact on the quality of life of patients on long-term renal replacement therapy.

Our results may suggest that it is worthwhile to attempt to use doxycycline to treat patients with dialysis-related amyloidosis, taking into account of drug’s low level of toxicity and its potential favourable antalgic effect in this difficult population. Further studies are needed to validate this potentially interesting option in dialysis related amyloidosis.

## Abbreviations

FDG: Fluorodexoxyglucose
Kt/V Daugirdas-2: Kt/V according to Daugirdas
normalised-PCR: Normalised protein catabolic rate
PET: Positron emission tomography
SUV: Standardised uptake value
